# Association between *PHOX2B* gene rs28647582 T>C polymorphism and Wilms tumor susceptibility

**DOI:** 10.1042/BSR20192529

**Published:** 2019-10-18

**Authors:** Ao Lin, Wen Fu, Wenwen Wang, Jinhong Zhu, Jiabin Liu, Huimin Xia, Guochang Liu, Jing He

**Affiliations:** 1 Department of Pediatric Surgery, Guangzhou Institute of Pediatrics, Guangzhou Women and Children’s Medical Center, Guangzhou Medical University, Guangzhou 510623, Guangdong, China; 2 Department of Oncology, The Affiliated Wuxi No.2 People’s Hospital of Nanjing Medical University, Wuxi 214000, Jiangsu, China; 3 Department of Clinical Laboratory, Biobank, Harbin Medical University Cancer Hospital, Harbin 150040, Heilongjiang, China

**Keywords:** PHOX2B, polymorphism, rs28647582, susceptibility, Wilms tumor

## Abstract

Wilms tumor is one of the most common pediatric solid tumors. The *pair-like homeobox 2b* (*PHOX2B*) gene is an important transcription factor that regulates cellular proliferation and differentiation in early life. The association between *PHOX2B* single nucleotide polymorphisms (SNPs) and Wilms tumor risk has not been investigated. Therefore, we conducted a case-control study involving 145 Wilms tumor patients and 531 controls to explore the association between the *PHOX2B* rs28647582 T>C polymorphism and Wilms tumor susceptibility. The association between the *PHOX2B* rs28647582 T>C polymorphism and Wilms tumor susceptibility was assessed by odds ratios (ORs) and 95% confidence intervals (CIs). Our results indicated that *PHOX2B* rs28647582 T>C polymorphism did not significantly alter Wilms tumor susceptibility. However, in the stratified analysis, we found that TC/CC genotypes significantly increased Wilms tumor risk among children older than 18 months (adjusted OR = 1.77, 95% CI = 1.07–2.95, *P*=0.027) and those with clinical stages III+IV (adjusted OR = 1.75, 95% CI = 1.09–2.82, *P*=0.022), when compared with those with TT genotype. Our study suggested that *PHOX2B* rs28647582 T>C was weakly associated with Wilms tumor susceptibility. Our conclusions need further validation with a larger sample size.

## Introduction

Wilms tumor, also known as nephroblastoma, is one of the most common pediatric malignant tumors, accounting for 7–8% of tumors in childhood [1,2]. The prevalence of Wilms tumor in Chinese children is approximately 3.3/million [3]. According to the report, 75% of patients are younger than 5-years old and incidence peak is 3-year old [1,4]. Survival rate of Wilms tumor once was less than 30%. Benefitting by the combined utilization of surgery, chemotherapy, radiotherapy and other treatment methods, 90% of Wilms tumors patients can be cured nowadays [4]. But, approximately 25% of Wilms tumor patients still have a poor survival rate of less than 70%, which results from unfavorable histopathological types [2]. Even though these patients survive, they also have a high recurrence rate and suffer from chronic health problems. There are some theories that try to explain the origin of nephroblastoma. The more frequently accepted theory is that posterior renal blastocyst fail to differentiate into glomeruli and renal tubules [5]. However, the mechanism of unsuccessful differentiation of posterior renal blastocyst is unknown. Therefore, it is necessary to explore genetic etiology of Wilms tumor to provide theoretical basis for its prediction and treatment.

With the development of genome-wide association studies (GWASs), more and more Wilms tumor susceptibility genes have been discovered. For example, single nucleotide polymorphisms (SNPs) in the *FWT1* [6], *FWT2* [7], *BARD1* [8], *CTR9* [9], *HACE1* [10] and *LMO1* genes [11] have been observed to modify Wilms tumor susceptibility. There are plenty of gene polymorphisms worth further exploring.

The homeobox genes are transcription factors that play an important part in embryonic development, including cellular differentiation, migration, apoptosis, signal transduction and angiogenesis [12]. The *pair-like homeobox 2b* (*PHOX2B*) gene belongs to the homeobox gene family which locates in 4p13. This gene accounts for 4.8 k bases approximately, contains three exons and two introns, encodes a peptide containing 314 amino acids [13]. *PHOX2B* is expressed during neural development in central autonomic circuits and peripheral neural crest derivatives, particularly in retrotrapezoid nucleus, noradrenergic centers and hindbrain. *PHOX2B* is an indispensible transcription factor in the proliferation and differentiation of neural crest tissue [14–17]. It can also regulate the expression of cancer-related genes such as *TH* [18], *PHOX2B* itself [19], *RET* [20], *TLX-2* [21], *ALK* [22], *SOX10* [23] and *MSX1* [24].

In the past, research about *PHOX2B* polymorphisms mostly focused on exons. With the continuous development of biotechnology, many introns have been found to assume the regulation roles in genetic expression. Previous investigations have shown that the *PHOX2B* rs28647582 T>C polymorphism significantly alters Hirschsprung disease susceptibility [25–27]. In addition, Perotti et al. [28] and Zin et al. [29] have widely found the heterozygous loss of 4p13 in sporadic Wilms tumor. To our knowledge, no study has investigated the association between *PHOX2B* rs28647582 T>C polymorphism and Wilms tumor susceptibility. Considering this polymorphism may affect the differentiation of posterior renal blastocyst, we conducted this experiment about *PHOX2B* rs28647582 T>C polymorphism and Wilms tumor susceptibility.

## Materials and methods

### Study population

It was a case-control study with 145 patients and 531 controls (Supplementary Table 1). The patients were collected from the Guangzhou Women and Children’s Medical Center during March 2001 to June 2016 [30–32]. Wilms tumor patients were histopathologically confirmed and distinguished depending on the NWTS-5 criteria. At the same time, a total of 531 tumor-free controls were randomly selected from the same center and matched patients with age, gender and ethnicity. In accordance with the relevant laws and regulations, participants or their guardians were requested to sign informed consent. The present study was approved by the Institutional Review Board of Guangzhou Women and Children’s Medical Center (ethic approve number: 2018022102).

### Genotyping

DNA was extracted from 2 ml venous blood samples using TIANamp Blood DNA Kit (TianGen Biotech Co. Ltd., Beijing, China). The *PHOX2B* rs28647582 T>C polymorphism was genotyped by TaqMan real-time PCR [33–35]. The information of samples was kept secret from staffs for a reliably experimental results. Moreover, we randomly selected 10% of the samples for repetitive test. The results showed that repeatability is 100%.

### Statistical analysis

T-test was employed to check the differences in age. Frequency distribution of gender and genotype between patients and the controls were evaluated by χ^2^ test. A goodness-of-fit test was used to estimate Hardy–Weinberg equilibrium (HWE) in controls. After adjusting for age and gender, we performed an unconditional multiple logistic regression model to assess the association between the *PHOX2B* rs28647582 T>C polymorphism and Wilms tumor susceptibility by odds ratios (ORs) and 95% confidence intervals (CIs). Finally, stratified analysis was conducted by age, gender and clinical stages. All data were analyzed by SAS statistical software, using a two-sided test. The results were considered statistically significant when *P*<0.05.

## Results

### *PHOX2B* rs28647582 T>C polymorphism and Wilms tumor susceptibility

The genotype distribution of *PHOX2B* rs28647582 T>C polymorphism in Wilms tumor patients and controls is shown in Table 1. The genotype distribution of *PHOX2B* rs28647582 T>C polymorphism obeyed the HWE genetic balance in controls (*P*=0.505). Unfortunately, we could not observe significant association between *PHOX2B* rs28647582 T>C polymorphism and Wilms tumor susceptibility (TC vs. TT: adjusted OR = 1.42, 95% CI = 0.95–2.12, *P*=0.090; CC vs. TT: adjusted OR = 0.78, 95% CI = 0.22–2.76, *P*=0.699; TC/CC vs. TT: adjusted OR = 1.35, 95% CI = 0.91–2.00, *P*=0.133; CC vs. TC/TT: adjusted OR = 0.71, 95% CI = 0.20–2.48, *P*=0.587; and C vs. T: adjusted OR = 1.22, 95% CI = 0.87–1.72, *P*=0.252).

**Table 1:** *PHOX2B* rs28647582 T>C polymorphism and Wilms tumor susceptibility

Genotype	Cases (N = 145)	Controls (N = 531)	P^*^	Crude OR (95% CI)	*P*	Adjusted OR (95% CI)^†^	*P*^†^
rs28647582 (HWE = 0.505)							
TT	95 (65.52)	380 (71.56)		1.00		1.00	
TC	47 (32.41)	136 (25.61)		1.38 (0.93–2.06)	0.113	1.42 (0.95–2.12)	0.090
CC	3 (2.07)	15 (2.82)		0.80 (0.23–2.82)	0.729	0.78 (0.22–2.76)	0.699
Additive			0.249	1.21 (0.85–1.70)	0.280	1.22 (0.87–1.72)	0.254
Dominant	50 (34.48)	151 (28.44)	0.158	1.33 (0.90–1.96)	0.159	1.35 (0.91–2.00)	0.133
Recessive	142 (97.93)	516 (97.18)	0.616	0.73 (0.21–2.55)	0.618	0.71 (0.20–2.48)	0.587
T	237 (81.72)	896 (84.37)		1.00		1.00	
C	53 (18.28)	166 (15.63)	0.279	1.21 (0.86–1.70)	0.279	1.22 (0.87–1.72)	0.252

* χ^2^ test for genotype distributions between Wilms tumor patients and controls.

† Adjusted for age and gender.

### Stratification analysis

We further conducted a stratified analysis by age, gender and clinical stages (Table 2). Stratified analysis revealed that TC/CC genotypes carriers had higher risk to develop Wilms tumor than those with TT genotype in the groups of older than 18 months (adjusted OR = 1.77, 95% CI = 1.07–2.95, *P*=0.027) and clinical stages III+IV (adjusted OR = 1.75, 95% CI = 1.09–2.82, *P*=0.022). No significant correlation was found between *PHOX2B* rs28647582 T>C polymorphism and Wilms tumor susceptibility in other groups.

**Table 2:** Stratification analysis for the association between *PHOX2B* rs28647582 T>C polymorphism and Wilms tumor susceptibility

Variables	rs28647582 (cases/controls)		Crude OR	*P*	Adjusted OR^*^	P^*^
	TT	TC/CC	(95% CI)		(95% CI)	
Age, month						
≤18	51/174	15/59	0.87 (0.45–1.66)	0.667	0.86 (0.45–1.65)	0.651
>18	44/206	35/92	**1.78 (1.07–2.96)**	**0.026**	**1.77 (1.07–2.95)**	**0.027**
Gender						
Females	45/164	19/69	1.00 (0.55–1.84)	0.991	1.02 (0.56–1.88)	0.945
Males	50/216	31/82	1.63 (0.98–2.73)	0.062	1.65 (0.98–2.76)	0.059
Clinical stage						
I+II	37/380	16/151	1.09 (0.59–2.02)	0.788	1.14 (0.61–2.12)	0.685
III+IV	49/380	34/151	**1.75 (1.08–2.81)**	**0.022**	**1.75 (1.09–2.82)**	**0.022**

* Adjusted for age and gender, omitting the corresponding factor. Results in bold indicate *P* <0.05.

## Discussion

*PHOX2B* mutations were observed in congenital central hypoventilation syndrome, neuroblastoma and Hirschsprung disease [36,37]. Subsequent studies confirmed that polyalanine repeat expansion mutation (PARMs) in exon 3 increased the risk of congenital central hypoventilation syndrome [38,39]. As for exon 3 NPARMs (exon 3 nucleotide 673 G>T and 702_714dup13) and mutations in other locations (exon 2 nucleotide 299 G>T and nucleotide 421 C>G), they were more likely to modify the susceptibility to familial and sporadic neuroblastoma [40,41].

Introns are widely distributed in the genome of eukaryotic cells. They are much longer than exons and accumulate more mutations, accounting for 95–97% in human genome. Although introns do not code for proteins, an increasing number of studies found that introns not only played a regulatory role in transcription, but also had selective splicing and promoter-like functions during mRNA maturation and translation [42]. *PHOX2B* rs28647582 is located in intron 2. Existing studies had no idea how rs28647582 affected *PHOX2B*. In the past few years, some conflicting results about *PHOX2B* rs28647582 polymorphism and Hirschsprung’s disease were drawn. Liu et al. [25] found that rs28647582 C allele increased Hirschsprung’s disease risk. But, Garcia-Barcelo et al. [26] and Xiao et al. [27] indicated that rs28647582 CC/CT were protective genotypes to Hirschsprung’s disease. Interestingly, a meta-analysis showed that the rs28647582 T>C polymorphism failed to significantly change Hirschsprung’s disease risk [43]. These researches suggested that rs28647582 polymorphism may modify certain diseases susceptibility.

This is the first investigation about *PHOX2B* rs28647582 T>C polymorphism and Wilms tumor. We observed that *PHOX2B* rs28647582 TC/CC genotypes significantly increased Wilms tumor susceptibility in subgroups older than 18 months or clinical stages III+IV. Based on previous studies [26,41], rs28647582 T>C polymorphism would change *PHOX2B* structure and expression by abnormally splicing and regulating, which would weaken *PHOX2B* ability to promote the proliferation and differentiation of renal cells. In addition, *PHOX2B* rs28647582 T>C polymorphism may combine with tumor-related genes to modify Wilms tumor susceptibility.

Although we found the correlation between *PHOX2B* rs28647582 T>C polymorphism and Wilms tumor susceptibility in stratified analysis, some limitations existed in our study. First, our research had a relatively small sample size, containing 145 patients and 531 controls. It was difficult to discover the genuine association between *PHOX2B* rs28647582 T>C polymorphism and Wilms tumor. Second, we selected only one polymorphism in *PHOX2B*, which made genotype association analysis impossible. It may omit the combined effect of *PHOX2B* rs28647582 T>C polymorphism and other potential polymorphisms in Wilms tumor [44]. Third, as a hospital-based retrospective study, hospital admission bias and information bias inevitably existed in population selection, information collection and experimental operation. Finally, this paper merely revealed the connection between *PHOX2B* rs28647582 T>C polymorphism and Wilms tumor susceptibility. In order to achieve reliable results, we should expand the sample size, add genotype association analysis and perform a strict quality monitoring in follow-up studies.

In conclusion, we have exposed that *PHOX2B* rs28647582 T>C polymorphism is weakly associated with Wilms tumor susceptibility. Our result need further verified.

## Supplementary Material

Supplementary Table S1

## Abbreviations

CI: confidence interval
GWAS: genome-wide association study
HWE: Hardy-Weinberg equilibrium
OR: odds ratio
*PHOX2B*: *pair-like homeobox 2b*
SNP: single nucleotide polymorphism
