# Supplementary material for: Association between PHOX2B gene rs28647582 T>C polymorphism and Wilms tumor susceptibility
Source: Biosci Rep. 2019 Oct 18;39(10):BSR20192529. doi: 10.1042/BSR20192529 (PMC6822530; doi:10.1042/BSR20192529)
Supplement: Supplementary Table S1 [file BSR-2019-2529_supp.pdf]

Supplemental Table 1. Frequency distribution of selected variables for Wilms tumor cases and cancer-free controls

Variables	Cases (n=145)		Controls (n=531)		P [a]
	No.	%	No.	%	
Age range, month	1-132		0.07-156		0.725
Mean ± SD	26.17 ± 21.48		29.73 ± 24.86		
≤18	66	45.52	233	43.88	
>18	79	54.48	298	56.12	
Gender					0.956
Female	64	44.14	233	43.88	
Male	81	55.86	298	56.12	
Clinical stages					
I	4	2.76			
II	49	33.79			
III	50	34.48			
IV	33	22.76			
NA	9	6.21			

SD, standard deviation; NA, not available.

[a] Two-sided χ^2 test for distributions between Wilms tumor cases and cancer-free controls.
